# Characterizing Continuous Glucose Monitoring Prescription Patterns Between 2020 and 2024: A Retrospective Study at a Single Primary Care Facility in the United States

**DOI:** 10.1155/jdr/8650831

**Published:** 2026-09-16

**Authors:** Rahma Mkuu, Protiva Rahman, Daniela Rivero-Mendoza, William Troy Donahoo, Elizabeth Shenkman, Diandra Ojo, Marcos R. Lamas

**Affiliations:** ^1^ Department of Health Outcomes & Biomedical Informatics, University of Florida, Gainesville, Florida, USA, ufl.edu; ^2^ Department of Medicine, University of Florida, Gainesville, Florida, USA, ufl.edu; ^3^ Department of Electrical & Computer Engineering, University of Florida, Gainesville, Florida, USA, ufl.edu

## Abstract

**Objective:**

Examine the factors associated with continuous glucose monitoring (CGM) device prescriptions in a primary care facility.

**Materials and Methods:**

We conducted a retrospective study of 1476 patients with Type 1 and Type 2 diabetes receiving care at a single primary care facility between 2020 and 2024. We examined the associations between receiving a CGM prescription and demographic characteristics, health factors, de‐identified physician information, and insurance status. We conducted univariate analysis using chi‐square tests. Multivariate logistic regression models accounted for multiple factors predicting receiving a CGM prescription.

**Results:**

The average CGM prescription rate per physician was less than 4%. Various factors were significantly associated with receiving a CGM prescription. Notably, younger patients on insulin, with Type 1 diabetes, and higher glycated hemoglobin (HbA1C) values were more likely to be prescribed a CGM device. Patients with nutritional and metabolic diseases or abnormal lab values were less likely to receive a CGM prescription.

**Conclusions:**

Our findings are similar to those of other CGM studies, which show that CGM prescriptions are significantly higher among patients who are younger and who have Type 1 diabetes. There is a need for future studies that aim to better understand CGM prescribing decision‐making among PCPs to inform the development of targeted interventions to improve CGM prescribing rates for all eligible patients.

## 1. Introduction

Optimizing glycemic levels through blood glucose monitoring is an important aspect of diabetes management [1, 2]. Continuous glucose monitoring (CGM) provides real‐time glucose level data compared with conventional fingerstick‐based self‐monitoring of blood glucose (SMBG), which only provides data at specific times of day [3]. In a study conducted across six primary care clinics, patients with a diagnosis of Type 2 diabetes who had been prescribed a CGM device had significantly greater HbA1c control compared with patients without a CGM device [4]. A systematic review that identified 10 studies that explored the use of CGM in primary care settings found that patients with a CGM device were more effective in managing glucose levels compared with those without a device [5]. Other advantages of CGM devices include the ability to predict the direction or rate of change of glucose levels, alerting patients when glucose levels are too low or high, allowing patients to share their data with providers, and cost reductions in hospitalizations due to hypoglycemia and diabetic ketoacidosis events ([6]; [7]; [8]; [9]; [10]). In fact, although CGM devices have a higher direct acquisition cost than traditional SMBG, studies suggest that CGM may be cost‐effective and, in some populations, may result in lower overall healthcare costs [11].

Research on the implementation of CGM has mostly focused on hospitalized or critically ill patients [12, 13]. In inpatient or hospital settings, CGM decreases the burden of frequent point‐of‐care testing for critically ill and hospitalized patients [14–16]. The use of CGM has expanded to the primary care setting with the support of CGM coverage [17, 18]. CGM use in primary care increased significantly between 2020 and 2021, with one large medical center in the Southeastern United States (US) reporting a 125% increase [19]. In the study, patients with private insurance or being treated by endocrinologists were more likely to use CGM compared with those being treated by primary care providers (PCPs) [19]. Of note, PCPs treat 90% of patients with diabetes because of the limited number of endocrinology specialists [11, 20, 21].

Despite the promise of CGM to improve diabetes management and outcomes, few studies have examined patterns of CGM prescribing, particularly in primary care settings. Studies highlight barriers that hinder the widespread implementation of CGM prescribing from patient and provider perspectives, including skin irritation, alarm fatigue, information overload, and cost ([7]; [8]; [9]). Insurance reimbursement is cited as the most significant barrier, as it precludes patients from accessing the device altogether, especially patients with limited access to specialists such as those on public insurance (e.g., Medicaid) [22]. Studies show that some patients, including Hispanic and Black populations [23], individuals with Type 2 diabetes [24] and those with noninsulin treatment [19] are less likely to be prescribed CGM. The studies, however, are scarce, and more research is needed to better understand CGM prescription patterns to inform implementation efforts to support CGM prescribing in primary care settings. This study is aimed at examining characteristics associated with receiving a CGM prescription among primary care patients with diabetes.

## 2. Methods

### 2.1. Study Population

We analyzed data from a primary care clinic affiliated with a large academic medical center in North Central Florida. Patients who received care from January 1, 2020, to August 31, 2024, were included in our cross‐sectional study. Deidentified data from the systems′ Integrated Data Repository included demographic information (e.g., race, gender, date of birth, health insurance), health conditions including diabetes type, prescriptions including CGM prescription, and laboratory results. We included patients 18 years or older with a diabetes diagnosis (*n* = 1497). We removed one patient with missing insurance information and 23 patients with unspecified diabetes type (*n* = 1473).

### 2.2. Variables

#### 2.2.1. Outcome Variable

The outcome variable was receipt of a CGM prescription. We received deidentified dates and physician identifiers for the CGM prescription. We had an additional table with CGM procedure dates. If a patient was listed in the CGM prescription table or the CGM procedures table, we marked their CGM status as having received a CGM prescription (1). If they were not listed in either table, their status was marked as zero, indicating that they did not have a CGM prescription. The CGM date was defined as the earliest CGM prescription or procedure date. In our cohort of 1473 patients, 328 had CGM.

#### 2.2.2. Control Variables

Demographic characteristics including sex (female, male), race (Asian, Black, other, White [ethnicity information was not available]), and age in years. We grouped insurance statuses into private, Medicaid, Medicare, and Dufexeal‐Enrolled (having both Medicare and Medicaid). We used patients′ most recent diabetes diagnosis ICD10 code to decide if they were Type 1 or Type 2. Additionally, Type 1 diabetes patients were required to have an insulin prescription. We used the earliest diabetes diagnosis as the date of confirmed diabetes to minimize confounders from relying on the date a patient received insulin treatment as an earliest diagnosis date. For diabetes treatment, patients who did not receive insulin but other treatments, such as insulin‐sensitizing agents, were categorized as having noninsulin diabetes treatment. We counted the number of inpatient visits prior to diabetes diagnosis as a proxy for chronic illnesses and whether that might affect CGM administration. The higher number of visits is associated with more chronicity, such as having multiple chronic conditions [25, 26]. For comorbidities, we used the diagnosis codes [27] in the problem list [28], which were rolled up to broader categories to increase power. Comorbidities before the confirmed diabetes diagnosis were included and present in at least 10% of the population. For the HbA1C score, we used the most recent year available for each patient and reported the mean of all readings taken in that year. Time to treatment was measured as time from confirmed diabetes diagnosis to any treatment—insulin or noninsulin.

#### 2.2.3. Physician Prescribing Pattern

**Figure 1:**
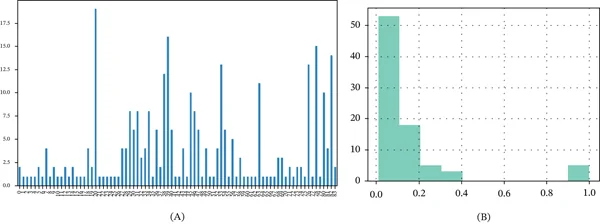
Physician prescription patterns in a single primary facility between 2020 and 2024. (A) number of CGM prescriptions per Physician. (B) histogram of rate of physician prescriptions.

We also analyzed outpatient visit data that included deidentified physician identifiers and patient visit dates. We matched CGM prescriptions by joining physician and patient identifiers in the CGM table to physician outpatient visit data. We calculated the rate of prescriptions by physician as the number of unique patients prescribed with a CGM by the physician divided by the total number of diabetic patients seen by the physician. We plotted a histogram of the rates of CGM prescription for physicians in Figure 1.

### 2.3. Statistical Analysis

#### 2.3.1. Univariate Analysis

Univariate Analysis summarized the characteristics of the patient population by whether the patient received a CGM prescription. *p*‐values for the univariate association between each covariate and CGM prescription are reported using the chi‐statistic [29]. We calculated CGM rates stratified by Type 1 and Type 2 diabetes and demographic factors such as race, age, and insurance type. We report the mean age and its association with CGM prescription. We also plot age alongside CGM prescription, stratifying age into broad categories of 18–40, 41–50, 51–65, and 65 or above (Figure 1). Rates were calculated as the number of people in that group with a CGM prescription divided by the total number of people in the group.

#### 2.3.2. Multivariate Analysis

We performed multivariate logistic regression using factors that were significant in the univariate analysis (see Table 1) to predict CGM device prescription using Python′s statsmodel package [30]. These factors included insurance, diabetes type, diabetes treatment, age at diagnosis, time to treatment, HbA1C value, abnormal lab values (e.g., impaired fasting glucose, persistent proteinuria), and nutritional or metabolic disease (e.g., hyperlipidemia, vitamin D deficiency, morbid obesity). See Table S1 for descriptive statistics on the number of patients with metabolic disorders or abnormal laboratory results. Diabetes treatment was a categorical variable (0 = no treatment, 1 = noninsulin treatment, and 2 = insulin treatment). Patients with missing values in any of these categories were excluded, resulting in 1265 patients. We split the dataset into 80:20 train and test sets. Thus, we constructed the model and retrieved coefficients using 80% of the dataset. We then evaluated the model by reporting the accuracy and area under the curve (AUC) on the unseen test set, to ensure robust results (Jin [31]).

**Table 1:** Descriptive statistics by continuous glucose monitoring prescribing.

		Overall (*n* = 1474)	No CGM (*n* = 1145)	CGM Prescribed (*n* = 328)	*p*
Sex, *n* (%)	Female	861 (58.5)	670 (58.5)	191 (58.2)	0.977
	Male	612 (41.5)	475 (41.5)	137 (41.8)	
Race, *n* (%)	Asian	109 (7.4)	86 (7.5)	23 (7.0)	0.689
	Black	267 (18.1)	206 (18.0)	61 (18.6)	
	Other	109 (7.4)	80 (7.0)	29 (8.8)	
	White	988 (67.1)	773 (67.5)	215 (65.5)	
Age at Diagnosis, mean (SD)		60.8 (13.2)	62.1 (12.7)	56.0 (13.7)	< 0.001
Insurance, *n* (%)	Dual‐enrolled	104 (7.1)	74 (6.5)	30 (9.1)	< 0.001
	Medicaid	89 (6.0)	62 (5.4)	27 (8.2)	
	Medicare	738 (50.1)	607 (53.0)	131 (39.9)	
	Private	542 (36.8)	402 (35.1)	140 (42.7)	
Diabetes type, *n* (%)	Type 1 diabetes	56 (3.8)	16 (1.4)	40 (12.2)	< 0.001
	Type 2 diabetes	1417 (96.2)	1129 (98.6)	288 (87.8)	
Diabetes treatment, *n* (%)	No treatment	202 (13.7)	196 (17.1)	6 (1.8)	< 0.001
	Insulin treatment	879 (59.7)	791 (69.1)	88 (26.8)	
	Noninsulin treatment	392 (26.6)	158 (13.8)	234 (71.3)	
A1C, mean (SD)		6.9 (1.5)	6.7 (1.3)	7.7 (1.7)	< 0.001
In patient visits, mean (SD)		54.4 (99.6)	57.1 (96.4)	44.6 (110.1)	0.087
Diseases of musculoskeletal system, *n* (%)	0	1286 (87.3)	989 (86.4)	297 (90.5)	0.056
	1	187 (12.7)	156 (13.6)	31 (9.5)	
Nutritional and metabolic diseases, *n* (%)	0	1327 (90.1)	1019 (89.0)	308 (93.9)	0.012
	1	146 (9.9)	126 (11.0)	20 (6.1)	
Diseases of circulatory system, *n* (%)	0	1286 (87.3)	989 (86.4)	297 (90.5)	0.056
	1	187 (12.7)	156 (13.6)	31 (9.5)	
Abnormal clinical and laboratory findings, *n* (%)	0	1271 (86.3)	975 (85.2)	296 (90.2)	0.023
	1	202 (13.7)	170 (14.8)	32 (9.8)	
Time to treatment, mean (SD)		3.3 (7.8)	3.6 (8.1)	2.5 (6.9)	0.026

This study was approved by the Institutional Review Board as an exempt study given the use of deidentified secondary data. This study was supported by the UF Gatorade Royalties.

## 3. Results

### 3.1. Univariate Analysis

Table 1 shows that the rate of CGM prescribing was significantly higher among younger patients, patients with Type 1 diabetes, and patients with insulin prescriptions. The mean age at diabetes diagnosis of patients prescribed with CGM devices is 62.5 years. Patients on Medicare are less likely to be prescribed a CGM device. Additionally, patients with nutritional and metabolic diseases or abnormal lab findings were less likely to be prescribed a CGM device.

**Figure 2:**
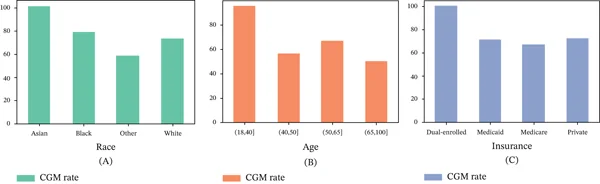
Continuous glucose monitoring rates (%) by (A) race, (B) age, and (C) insurance status for Type 1 diabetes patients in a single primary facility between 2020 and 2024.

After stratification by diabetes type, 56 patients had Type 1 diabetes. Forty of these patients were prescribed a CGM, at a rate of 71%. Figure 2 shows the CGM prescription rates in Type 1 diabetes patients with insulin, stratified by age, race, and insurance status. CGM prescription rates were highest among younger patients (i.e., those below 40 years old) and higher in Asian and Black patients. Further, the rate was the lowest in the Medicare category. The power was low in each category, preventing further examination of statistical differences.

### 3.2. Physician Prescription Patterns

Figure 1 shows physician prescriptions. Figure 1A shows the raw number of patients prescribed with CGM by the physicians, which ranges from 0 to 25. Figure 1B is a histogram of the prescription rates. The *x*‐axis is the prescription rate, and the *y*‐axis is the number of physicians. Most physicians had a CGM prescribing rate lower than 2%. One physician had a 100% rate, but this physician only saw one patient in our dataset.

**Figure 3:**
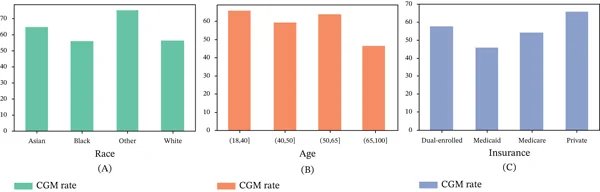
Continuous glucose monitoring rates (%) by (A) race, (B) age, and (C) insurance status for Type 2 diabetes patients in a single primary facility between 2020 and 2024.

There were 1417 patients with Type 2 diabetes. Of the patients not on insulin (*n* = 1081), 8.7% (*n* = 94) had a CGM prescription. Of the remaining patients (*n* = 336), 194 (57%) were on insulin. Figures 2 and 3 show the CGM prescription rates stratified by age, insurance, and race in Type 2 diabetes patients on insulin. CGM prescription rates were highest in younger patients and those with private insurance, but only the difference in age was statistically significant.

### 3.3. Multivariate Analysis

**Figure 4:**
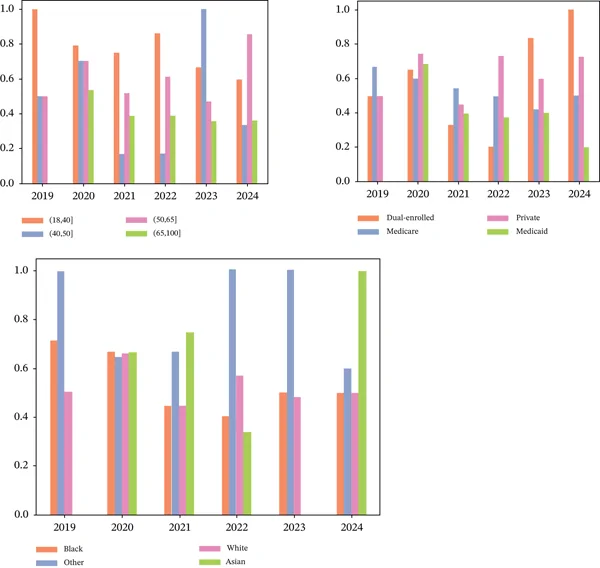
Trends in CGM prescribing across years by demographic characteristics.

Figure 4 provides a plot of CGM prescription trends by age and insurance status by year which were significantly associated with CGM prescription, Figure 4 also includes CGM prescription rates by race for each study year. The multivariate logistic regression had a predictive accuracy of 0.81 and an AUC of.76 on the test set. Table 2 shows the odds ratios with the 95% confidence intervals. Similar to the univariate case, age, and comorbidities were inversely associated with CGM prescription, as was being Black, whereas higher HbA1C and insulin were associated with a higher rate of CGM prescription.

**Table 2:** Regression coefficients, multivariate regression analysis in a single primary facility between 2020 and 2024.

	No CGM	CGM	OR
Age at diagnosis	0.96	0.99	0.98
Race Black	0.29	1.05	0.55
Race Asian	0.43	1.95	0.91
Race White	0.45	1.4	0.79
Diabetes type	0.32	1.2	0.62
Diabetes treatment	0.15	3.42	0.72
Nutritional and metabolic diseases	0.36	1.31	0.69
Abnormal clinical and laboratory findings	0.45	1.4	0.8
Insurance Medicaid	0.26	1.38	0.6
Insurance Medicare	0.54	1.81	0.98
Time to treatment	0.97	1.02	0.99
Insurance private	0.56	2.01	1.06
A1C	0.96	1.18	1.07
Insulin	3.21	84.63	16.49

## 4. Discussion

We examined CGM prescription patterns at an academic medical center′s primary care clinic located in North Central Florida. We found CGM prescribing discrepancies by demographic characteristics and diabetes type, supporting research from previous studies reporting CGM prescribing to be higher among some demographic groups [23, 32] and higher among patients with Type 1 diabetes [24]. The differential CGM prescribing patterns need to be addressed in order to ensure all patients can benefit from the technology that has been shown to improve diabetes management and outcomes. Given that the majority of patients with diabetes are treated by PCPs, there is a need for a better understanding of CGM prescription decision‐making among PCPs to inform future interventions targeted at promoting CGM prescription across all patients among PCPs [33].

Demographic and health factors, including younger age, use of insulin, Type 1 diabetes, and HbA1C values, were associated with a higher likelihood of being prescribed a CGM device. Having a nutritional and metabolic disease or abnormal lab values was associated with a lower likelihood of receiving a CGM prescription. It is possible that patients with other complex comorbidities are not prescribed CGM devices.

In our study, the CGM prescription rates were not statistically different by race. The rates of CGM prescription were around 22% across different races. Prior studies report that Hispanic and Black populations have a lower likelihood of receiving a CGM prescription compared with White adults [23, 32]. Kanbour et al. found that Black patients were less likely to have discussions about CGMs with their health provider and less likely to be prescribed subsequent CGM use [32]. Our findings may differ from other research because the clinic from which the data were derived serves the majority of the White population.

In our study, younger patients were more likely to be prescribed with CGM, which has been corroborated in prior studies by Mayberry [19] and Morrow [34]. A narrative review of trials between 1994 and 2004, comparing standard blood glucose monitoring (SBGM) and CGM in adults over 65 with diabetes, found that CGM use reduced hypoglycemia, improved well‐being, and quality of life [35]. The higher distrust of technology in older adults might be a barrier to CGM prescription for that group [36]. There is thus a need for interventions designed to educate the older adult population on these devices. Physicians serving this population would also need training and support in discussing these devices. The higher number of comorbidities in older adults would also pose a barrier to getting insurance approval for CGM devices. Patients with Type 1 diabetes had a CGM prescription rate (61%), compared with those with Type 2 diabetes (57%). Similar findings were reported by Karter et al. [24]. One reason may be that providers perceive individuals with Type 2 diabetes as not needing continuous monitoring compared with individuals with Type 1 diabetes, who may be at higher risk of hypoglycemia and hyperglycemia. Our results show that the differences persist even when comparing patients treated exclusively in primary care. Mayberry′s study also found that 72% of CGM users were on insulin [19] which is very similar to our study, where 71% of those on CGM are also on insulin. This may be due to providers′ perception that individuals on insulin regimes can benefit more from medication adjustments following CGM remote measurements. Being on insulin is also a requirement for insurance authorization for a CGM prescription [37]. Similarly, we found that individuals with higher HbA1C were more likely to get a CGM prescription. Studies examining the relationship between CGM prescriptions and glycemic control in young adults have shown that those using a CGM tend to have significantly lower HbA1c levels compared with those who do not use one [4, 36], indicating that CGM can be an effective tool for achieving glycemic control.

Medicare patients were significantly less likely to receive CGM prescriptions in the univariate model but this was not statistically significant in the multivariate model. Oser et al. found that clinicians who served more Medicare patients reported more favorable attitudes toward future prescribing and higher confidence using CGM to manage diabetes than clinicians with lower Medicare patient volume [38]. In 2017, Medicare approved coverage of CGM devices for individuals with Type 1 diabetes, setting a precedent that prompted other insurance providers to follow their lead [39, 40]. Oser et al. suggest an increase in resources to assist physicians, particularly with regard to handling insurance issues, as a means for improving CGM access [38]. Morrow et al. findings are comparable to ours, reporting age, socioeconomic factors, and insulin as the most predictive factors for CGM use [34]. Although Morrow et al. looked at behavioral and psychological factors such as diabetes distress, our data shows an inverse relationship between nutritional and metabolic disorders and abnormal lab findings and CGM prescription [34].

Despite our study′s contribution to the literature about the distribution of CGM prescriptions among primary care patients, our study has some limitations. Our study is limited to one primary care clinic serving a largely White population; our predictive model has an AUC of 77, which indicates above average discrimination; however, it is meant to study significant factors and is not necessary for clinical decision support. The sample size was also small, therefore limiting generalizability. We validated our multivariable results on a held‐out test set, so the results should be generalizable for our institutional population. Further validation studies at other sites are needed for more robustness. Further, using this model for clinical decision support will reinforce prescriptions for younger patients while ignoring older adults. CGM adoption has barriers such as distrust of technology, sarcopenia, technology illiteracy, or device complexities, which might be exacerbated in older adults [41]. Other studies have mentioned the use of CGM in older adults and urged for clinical trials, and a clinical trial published in JAMA found a small but statistically significant improvement in older adults using CGM with Type 1 diabetes [42–44]. Although CGM delivers valuable glucose‐monitoring benefits, cost, clinical workflow integration, user comprehension, and psychosocial impacts create significant barriers that must be addressed [42, 43, 45].

## 5. Conclusion

We found that CGM device prescriptions are higher among younger, insulin‐using, Type 1 diabetes patients at primary care clinics. We also found that patients with nutritional/metabolic disorders or abnormal lab findings are less likely to receive a CGM prescription. There is a need for studies to understand decision‐making related to CGM prescribing patterns in primary care, to inform future interventions that support physicians to prescribe CGM to all eligible patients.

## Author Contributions

Rahma Mkuu: conceptualization, resources, investigation, supervision. Protiva Rahman: formal analysis, methodology, writing original draft, visualization. Daniela Rivero‐Mendoza: Project administration, writing, review and editing. William Troy Donahoo: Funding acquisition. Elizabeth Shenkman: Resources, writing, review, and editing. Diandra Ojo; Writing original draft. Marcos R. Lamas: Conceptualization, investigation, methodology.

## Funding

Research reported in this publication was supported by the University of Florida Gatorade Royalties and the National Cancer Institute of the National Institutes of Health, 10.13039/100000002, under Award Number K01CA292583.

## Disclosure

All authors read and approved the final manuscript. The content is solely the responsibility of the authors and does not necessarily represent the official views of the National Institutes of Health.

## Ethics Statement

The authors have nothing to report.

## Conflicts of Interest

The authors declare no conflicts of interest.

## Supporting information


**Supporting Information** Additional supporting information can be found online in the Supporting Information section. Data S1: Number of patients by the categories that contribute to the variables “metabolic disorders” and “abnormal laboratories.”

## Data Availability

The data that support the findings of this study are available from UF Health . Restrictions apply to the availability of these data, which were used under license for this study. Data are available from (https://idr.ufhealth.org/research‐services/research‐data/) with the permission of UF Health.
