# Supplementary material for: Characterizing Continuous Glucose Monitoring Prescription Patterns Between 2020 and 2024: A Retrospective Study at a Single Primary Care Facility in the United States
Source: J Diabetes Res. 2026 Sep 16;2026:8650831. doi: 10.1155/jdr/8650831 (PMC13579378; doi:10.1155/jdr/8650831)
Supplement: Supplementary file 1 [file JDR-2026-8650831-s001.docx]

**Appendix**

**Table 1: Number of Patients by the Categories that Contribute to the Variables “Metabolic Disorders” and “Abnormal Laboratories.”**

| **Diagnosis Description** | **Count** |
| --- | --- |
| Pure hypercholesterolemia, unspecified | 4 |
| Unspecified severe protein-calorie malnutrition | 4 |
| Hypomagnesemia | 5 |
| Iron deficiency | 8 |
| Mixed hyperlipidemia | 13 |
| Obesity, unspecified | 15 |
| Deficiency of other specified B group vitamins | 15 |
| Morbid (severe) obesity due to excess calories | 19 |
| Vitamin D deficiency, unspecified | 20 |
| Hyperlipidemia, unspecified | 46 |
| Headache, unspecified | 8 |
| Hyperglycemia, unspecified | 9 |
| Palpitations | 9 |
| Unspecified abdominal pain | 9 |
| Syncope and collapse | 9 |
| Impaired fasting glucose | 9 |
| Chest pain, unspecified | 11 |
| Shortness of breath | 13 |
| Dizziness and giddiness | 13 |
| Other specified abnormal findings of blood chemistry | 14 |
